# Are AI tutors ready for the TCM classroom? A multimodal evaluation of large language models across cognitive levels in Chinese medicine education

**DOI:** 10.3389/fmed.2026.1893231

**Published:** 2026-07-27

**Authors:** Jie Yang, Ruojia Wang, Fengying Guo, Yifan Chen

**Affiliations:** Department of Public Administration, School of Management, Beijing University of Chinese Medicine, Beijing, China

**Keywords:** artificial intelligence, large language models, medical education, multimodal analysis, Traditional Chinese Medicine

## Abstract

**Background:**

Large language models (LLMs) have shown promise in medical education, but most are trained primarily on Western medical data and lack sufficient exposure to Traditional Chinese Medicine (TCM). Despite growing interest in LLMs for TCM education, systematic evaluations of their performance remain limited. However, it remains unclear at which cognitive levels—from basic recall to clinical reasoning—these models can reliably support learners, and whether their multimodal capabilities meet the demands of authentic TCM pedagogical tasks.

**Objective:**

This study aimed to evaluate LLMs’ performance on authentic Chinese medicine knowledge tasks, including both text-based and image-based assessments, to provide evidence for their potential application in TCM education.

**Methods:**

Seven LLMs were evaluated across four task types: textual objective questions, textual subjective syndrome differentiation tasks, image-based objective herb identification, and image-based subjective tongue diagnosis. For objective tasks, performance was measured by accuracy. For subjective tasks, performance was evaluated using natural language processing metrics and blinded expert ratings. Statistical analyses included Cochran’s *Q* test, Friedman rank-sum test, and Bonferroni-corrected *post-hoc* comparisons. Inter-rater reliability was verified using the intraclass correlation coefficient.

**Results:**

Significant tiered performance differences emerged across 7 models. For textual objective questions, Doubao-1.5-Pro achieved the highest accuracy (91.23%), with performance declining markedly from lower-order memorization to higher-order clinical reasoning tasks. For textual subjective tasks, SparkDesk-v3.5 (94.11%), Doubao-1.5-Pro (93.03%), and Gemini-3-Preview (92.99%) formed the top tier. A notable subjective-objective performance inversion was identified: SparkDesk-v3.5 ranked lowest in objective accuracy but highest in subjective scoring. Our exploratory image-based results suggest that no model exceeded 60% accuracy in herbal identification (*N* = 55), while tongue diagnosis performance was comparatively higher but should also be interpreted cautiously (*N* = 223). Qualitative analysis using a four-category error typology revealed visual misidentification, cross-modal association failures, terminological-level errors, and reasoning-level inconsistencies.

**Conclusion:**

Top-tier large language models demonstrate potential as virtual teaching assistants for foundational Traditional Chinese Medicine knowledge navigation. However, their deployment in complex syndrome differentiation and multimodal teaching poses substantial risks due to model errors and insufficient clinical reasoning. Image-based findings should be considered preliminary and require validation in larger, more balanced multimodal datasets. Clearly defined applicability boundaries, rigorous manual review procedures, and continuous enhancement of domain-specific multimodal datasets are essential before integration into educational practice.

## 1 Introduction

LLMs have made significant breakthroughs and rapidly gained popularity in the fields of medical education and clinical decision support. Studies have demonstrated that top-tier general-purpose LLMs exhibit exceptional natural language understanding and medical knowledge reasoning capabilities across multiple authoritative medical benchmark tests (1, 2). For instance, models such as ChatGPT have already achieved scores exceeding the passing threshold in the highly challenging United States Medical Licensing Examination (USMLE) without specialized fine-tuning, empirically establishing the potential of LLMs as future medical “AI tutors” and decision support systems (3). This paradigm shift is reshaping how knowledge is acquired and clinical reasoning is trained in modern medical education (4).

Research in the field of TCM has also gained significant traction. Huang et al. (5) constructed a dataset containing 5,473 questions from the National TCM Practitioner Qualification Examination and developed the TCMBench evaluation benchmark, which provides a unified reference framework for performance comparison of LLMs in the TCM field. Liu et al. (6) selected 7 mainstream LLMs, evaluated LLMs using real-world clinical acupuncture cases, conducted a comparative evaluation against TCM specialists across five dimensions: Western medicine diagnosis, TCM syndrome differentiation, acupoint selection, acupuncture manipulation, and herbal prescription, and revealed differences in model performance on tasks requiring TCM clinical reasoning. Wang et al. (7) focused on the scenario of TCM postgraduate entrance examinations, systematically analyzed the auxiliary effects of LLMs in multiple educational components including practice problem-solving, concept explanation, and error analysis, and provided practical references for the intelligent development of TCM education.

However, there are still critical limitations to be addressed in existing studies. On the one hand, most available studies focus on clinical diagnosis and treatment scenarios, while systematic evaluations specifically targeting TCM education scenarios are markedly insufficient. On the other hand, the evaluation dimensions are relatively homogeneous, with most assessments centered on text-based knowledge question answering. There is a notable gap in evaluating model adaptability to multimodal content such as TCM tongue diagnosis and image-based Chinese herbal medicine identification, thus failing to comprehensively reflect the knowledge accuracy of models in real-world teaching scenarios (8).

Amid the ongoing digital transformation of TCM education, this study addresses a critical research gap with both theoretical and practical implications. This study provides empirical evidence to inform the integration of AI into personalized TCM education. AI-assisted instruction has been demonstrated to foster critical thinking among medical students by providing scaffolded learning environments. Previous research has indicated that the integration of AI-generated feedback into complex clinical decision-making training facilitates the identification of cognitive blind spots by learners (9). By performing a stratified assessment of AI performance across the Principle-Method-Formula-Medicine continuum, this study investigates the practical feasibility of AI as a “personalized teaching assistant” in reducing disparities in TCM educational resources and enhancing learners’ independent syndrome differentiation skills.

This study also elucidates the limitations of current mainstream LLMs in medical education. The tendency of LLMs to produce incorrect or fabricated outputs, including hallucination in the narrower sense of non-standard syndrome names or non-existent formulas, poses potential risks in healthcare settings (10). Through comprehensive cross-model evaluation, this study precisely delineates the capability boundaries of different models in TCM-related tasks, identifies the teaching contexts in which they can function as reliable “digital teaching assistants,” and specifies the clinical decision-making contexts where stringent safety safeguards must be implemented. This will help prevent the technological backsliding and educational misdirection that unregulated application may cause.

This study informs the development of domain-specific models and the refinement of fine-tuning strategies tailored to TCM educational contexts. By identifying the weaknesses of general-purpose large models in the Principle-Method-Formula-Medicine reasoning pipeline, this study provides clear directions for the future development of domain-specific large models that incorporate deep cultural context and authentic TCM clinical reasoning capabilities.

## 2 Materials and methods

**Figure 1:**
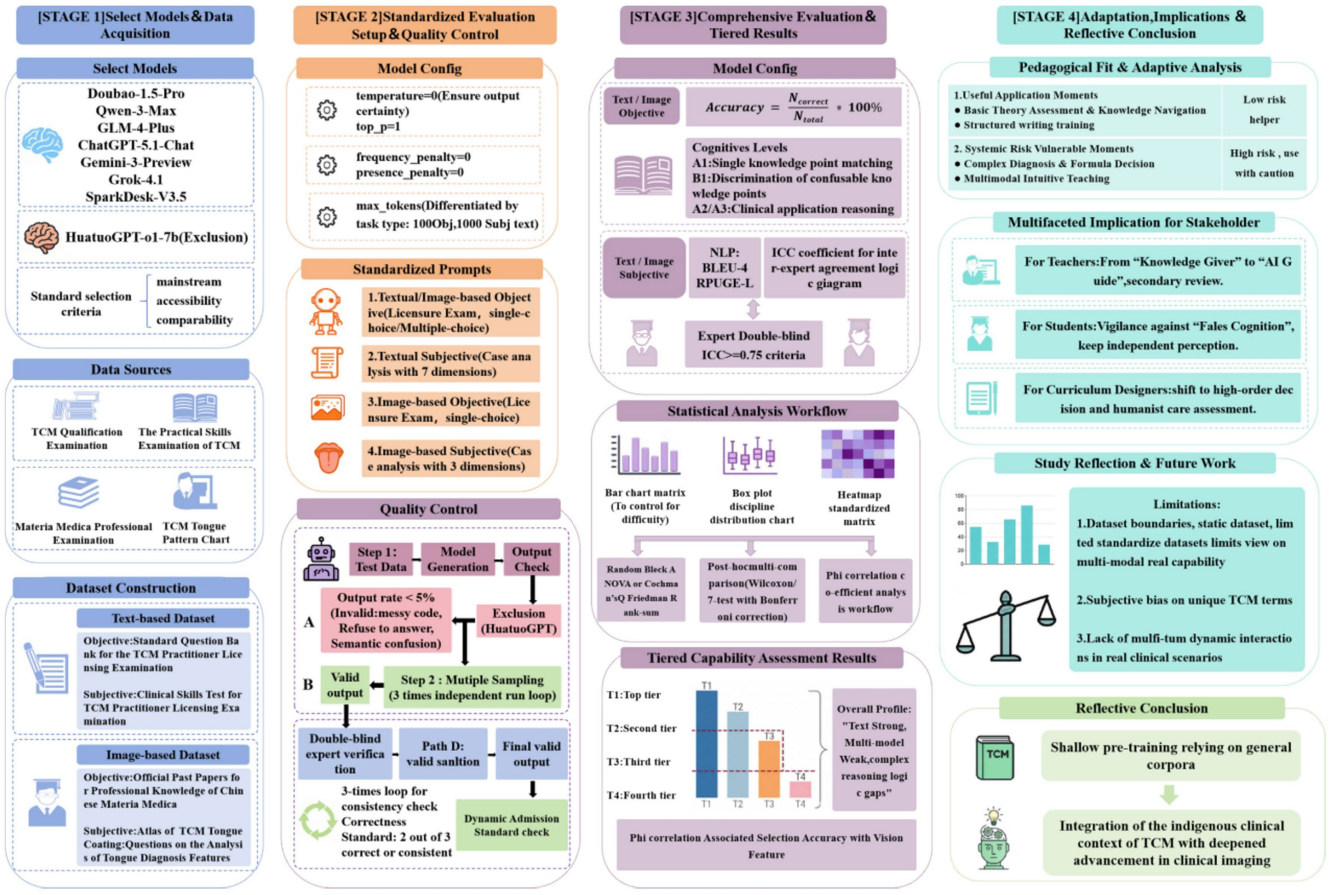
Technical roadmap.

The overall technical roadmap of this study is shown in Figure 1.

### 2.1 Study design and model selection

This study adopted a cross-sectional benchmarking design to evaluate the performance of mainstream domestic and international LLMs across text-based and image-based TCM educational tasks. All model evaluations were conducted between February 2026 and April 2026 under standardized prompting and parameter settings, so the findings should be interpreted as a snapshot of model performance during this testing window.

Models were selected based on representativeness, accessibility, and comparability to systematically evaluate the application performance of mainstream domestic and international LLMs in the field of TCM. The model inclusion criteria were as follows: ① industry-recognized first-tier general-purpose LLMs or representative domain-specific medical models; ② support API invocation or open-source deployment with parameter controllability; ③ demonstrate strong Chinese language comprehension and the ability to handle TCM-specific terminology.

The exclusion criteria were as follows: ① models with unstable API invocation, frequent output of garbled text, or systematic refusal to respond; ② models whose accuracy lagged significantly behind mainstream models or showed poor consistency during pre-testing; ③ models with severely outdated versions. Ultimately, 7 LLMs were finalized for in-depth evaluation (Table 1).

**Table 1:** Final selected models.

Serial No.	Model name	Category	Version	Release date	API provider
1	Doubao	Domestic General Large Model	1.5-Pro	October 2025	ByteDance
2	Qwen	Domestic General Large Model	3-Max	November 2025	Alibaba Group
3	GLM	Domestic General Large Model	4-Plus	September 2025	Zhipu AI
4	ChatGPT	Foreign General Large Model	5.1-Chat	January 2026	OpenAI
5	Gemini	Foreign General Large Model	3-Preview	February 2026	Google LLC
6	Grok	Foreign General Large Model	4.1	January 2026	xAI Corp
7	SparkDesk	Medical Vertical Model	v3.5	August 2025	iFLYTEK

### 2.2 Datasets

The dataset was constructed in compliance with medical education evaluation standards, covering four major question types: textual objective questions, textual subjective questions, image-based objective questions, and image-based subjective questions. It spans core competency dimensions including TCM theoretical knowledge, clinical application, and multimodal recognition.

#### 2.2.1 Textual objective questions

Textual objective questions are the most widely used question type for evaluating models’ mastery of basic TCM theories and clinical knowledge. The data were sourced from authentic questions of the National TCM Practitioner Qualification Examination from 2016 to 2025, covering 12 subjects with a total of 7,903 questions. The subjects include Basic Theories of TCM, TCM Diagnostics, Chinese Materia Medica, Prescriptions of TCM, Acupuncture and Moxibustion, TCM Internal Medicine, TCM Surgery, TCM Gynecology, TCM Pediatrics, TCM Classics, Health Regulations, and Medical Ethics. Among them, there are 5,239 Type A1 questions (single knowledge point memorization questions, lower-order cognition), 1,573 Type B1 questions (confusable knowledge point discrimination questions, middle-order cognition), 876 Type A2 questions (simple case syndrome differentiation questions, higher-order cognition), and 215 Type A3/A4 questions (complex clinical scenario questions, higher-order cognition). Selected examples are presented in Table 2.

**Table 2:** Question types of the Traditional Chinese Medicine practitioner qualification examination.

Subject	Question type	Question content
Prescriptions of Chinese Medicine	A1	Which of the following is NOT included in the “Eight Therapeutic Methods”? A. Diaphoresis, Emesis B. Purgation, Clearing C. Ventilation, Unblocking D. Clearing, Tonification E. Harmonization, Warming
Acupuncture and Moxibustion	B1	What is the distribution rule of the three Yin meridians of the foot below 8 cun above the medial malleolus? What is the distribution rule of the three Yin meridians of the foot above 8 cun above the medial malleolus? A. Jueyin anterior, Taiyin middle, Shaoyin posterior B. Shaoyin anterior, Jueyin middle, Taiyin posterior C. Jueyin anterior, Shaoyin middle, Taiyin posterior D. Taiyin anterior, Jueyin middle, Shaoyin posterior E. Taiyin anterior, Shaoyin middle, Jueyin posterior
Chinese Materia Medica	A2	Patient, female, 50 years old. Presents with chest tightness and suffocation, profuse yellow sticky sputum, and dry stool. Which of the following is the first-choice drug? A. Fructus Aurantii Immaturus B. Fructus Trichosanthis C. Ramulus Cinnamomi D. Radix Curcumae E. Rhizoma Chuanxiong
Internal Medicine of Traditional Chinese Medicine	A3	Patient, male, 25 years old. Has had fever for 2 days. Presents with severe aversion to cold, fever, no sweating, headache and body ache, cough with white sputum, weak expectoration. The patient is usually fatigued and weak, short of breath and reluctant to speak, and prone to repeated colds. Tongue is pale with white coating, pulse is floating and weak. What is the syndrome pattern? A. Wind-cold fettering the exterior B. Wind-heat attacking the exterior C. Yin-deficiency common cold D. Qi-deficiency common cold E. Summer-dampness damaging the exterior

#### 2.2.2 Textual subjective questions

Textual subjective questions are designed to evaluate the models’ end-to-end capability in TCM clinical syndrome differentiation and treatment. The data were sourced from authentic medical case analysis questions of the National TCM Practitioner Qualification Examination (Practical Skills Test) from 2016 to 2025, with a total of 115 questions. Examples of the questions are shown below:

*Patient Qian, male, 44 years old, married, farmer. First consultation on November 19, 2015. The patient had experienced recurrent paroxysmal wheezing and dyspnea with phlegm in the throat since childhood, and was prone to colds, spontaneous sweating, and aversion to wind in daily life. One month ago, wheezing recurred after catching a cold, accompanied by cough and dyspnea, which had improved after treatment. Current symptoms: Shortness of breath and low voice, occasional mild wheezing in the throat, profuse thin white sputum, lassitude and weakness, spontaneous sweating, aversion to wind, poor appetite and loose stools, pale tongue with white coating, thready and weak pulse. Please differentiate it from Dyspnea Syndrome.*

#### 2.2.3 Image-based objective questions

**Figure 2:**
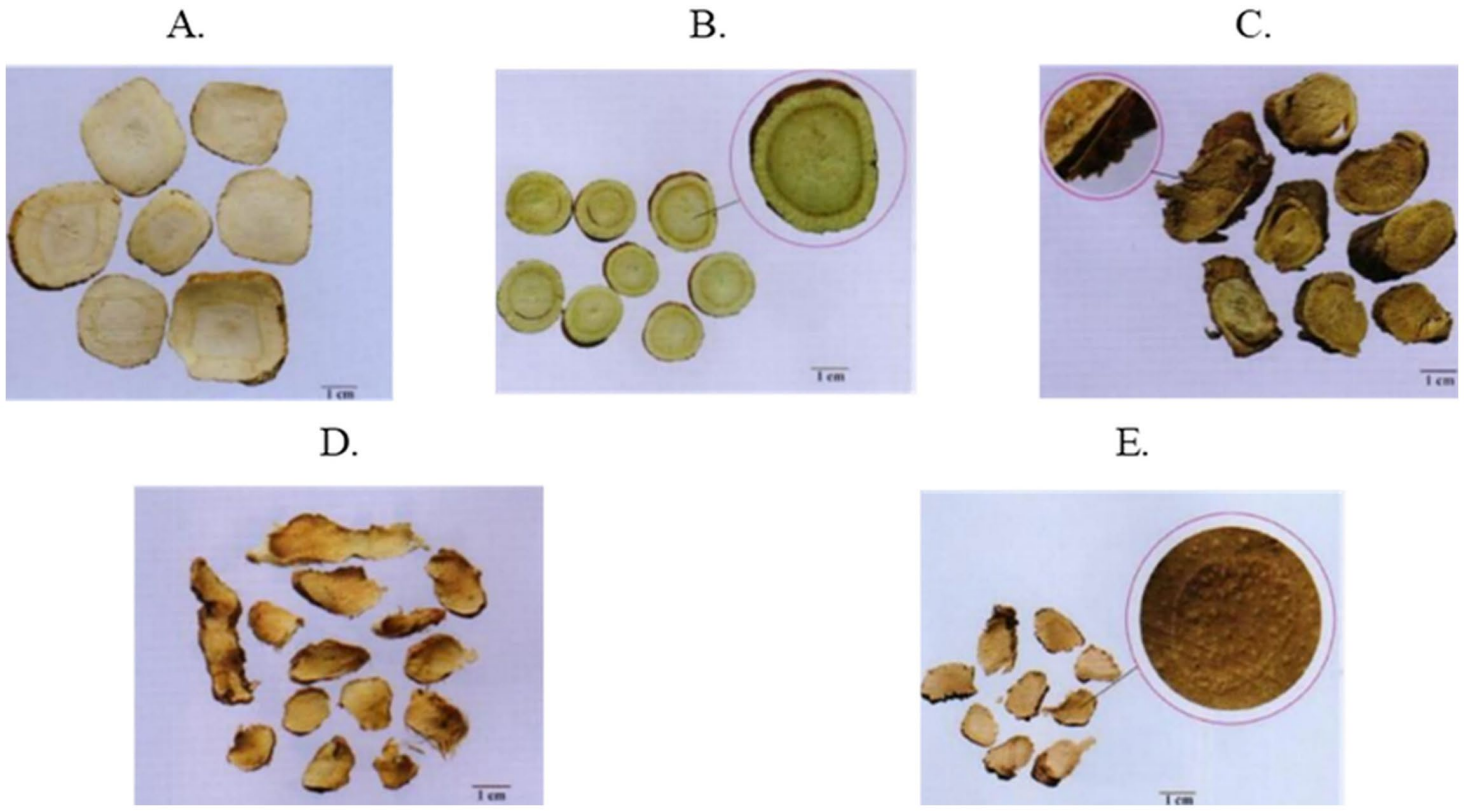
Example of a picture-based drug identification question. **(A)** Pale irregular root slices; **(B)** greenish-yellow circular slices with an enlarged cross-section; **(C)** dark-brown irregular pieces with an enlarged surface inset; **(D)** thin curled yellow-brown slices; and **(E)** small tan fragments with an enlarged view of brown powder.

Image-based objective questions focus on evaluating the models’ capability of Chinese medicinal herb identification. The data were sourced from image-based medicinal herb identification multiple-choice questions extracted from authentic questions of the National Licensed Pharmacist Examination from 2015 to 2025, with a total of 55 questions. Examples of the questions are shown in Figure 2.

#### 2.2.4 Image-based subjective questions

**Figure 3:**
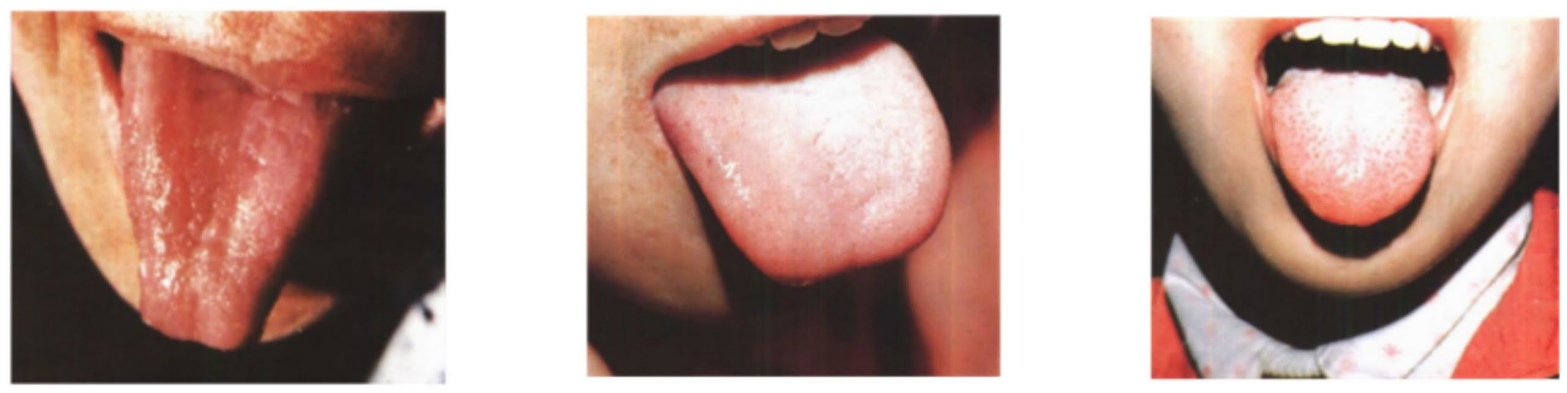
Example of subjective questions in tongue diagnosis. Please describe the tongue manifestation in the figure (including the characteristic of tongue color, tongue morphology, and tongue coating), as well as its corresponding main clinical conditions and syndrome differentiation.

Image-based subjective questions focus on evaluating the models’ in-depth interpretation of TCM-specific characteristic images. The data were sourced from classic tongue diagnosis image cases in Atlas of Tongue Coating in Traditional Chinese Medicine, an authoritative reference in the field of TCM teaching, with a total of 223 questions. Examples of the questions are shown in Figure 3.

### 2.3 Evaluation process

#### 2.3.1 Model parameter configuration

To ensure results were consistent and reproducible, the generation parameters of all models were uniformly set as follows: temperature = 0 (disabling stochastic sampling to guarantee output determinism), top_p = 1, frequency_penalty = 0, presence_penalty = 0. The max_tokens parameter was set differentially according to question types: it was limited to 100 tokens for objective questions to minimize redundant output, set to 1,000 tokens for textual subjective questions to ensure the integrity of responses, and limited to 300 tokens for image-based subjective questions to guarantee concise analysis.

#### 2.3.2 Prompt design

**Figure 4:**
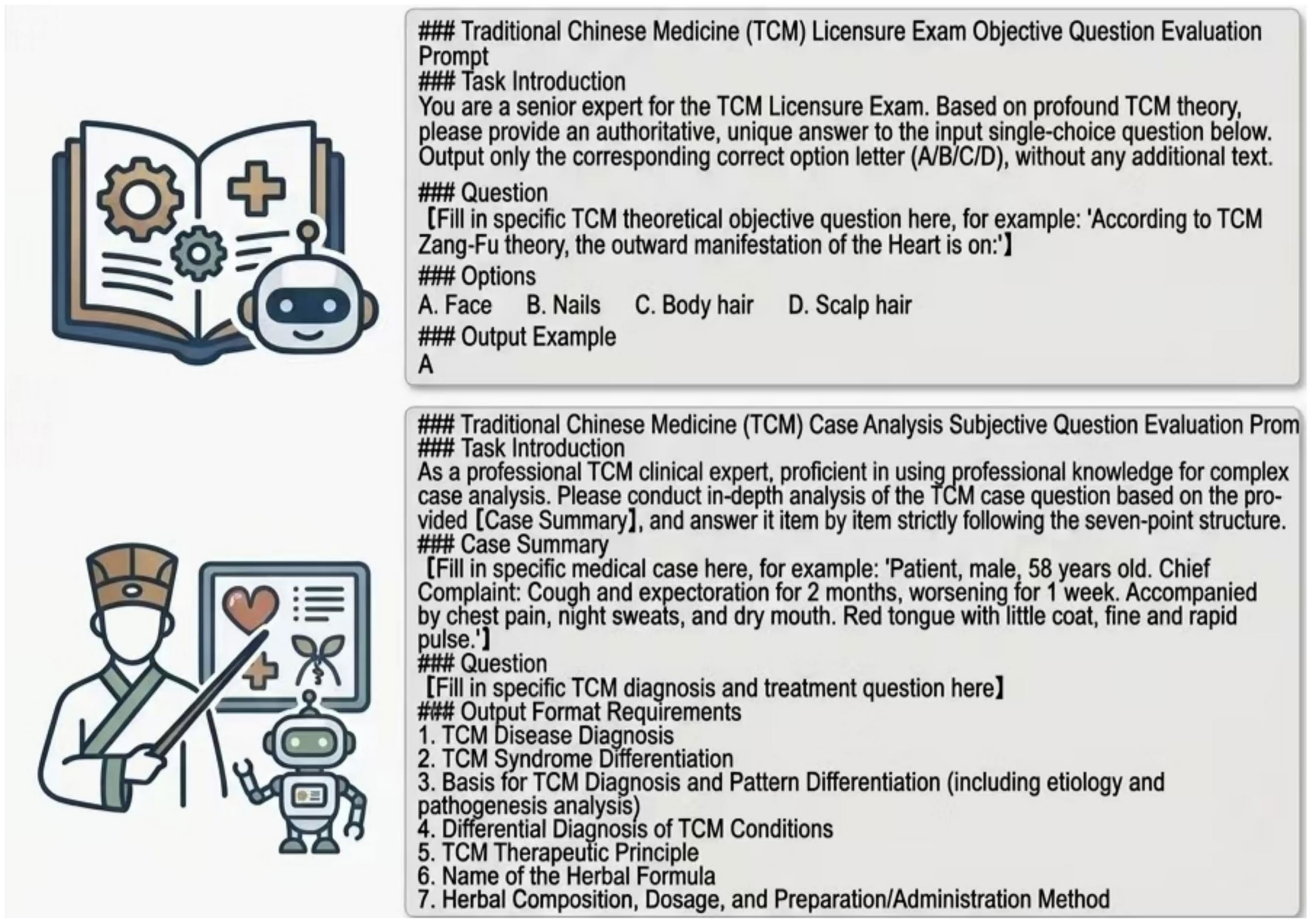
Design of text-based question prompts.

**Figure 5:**
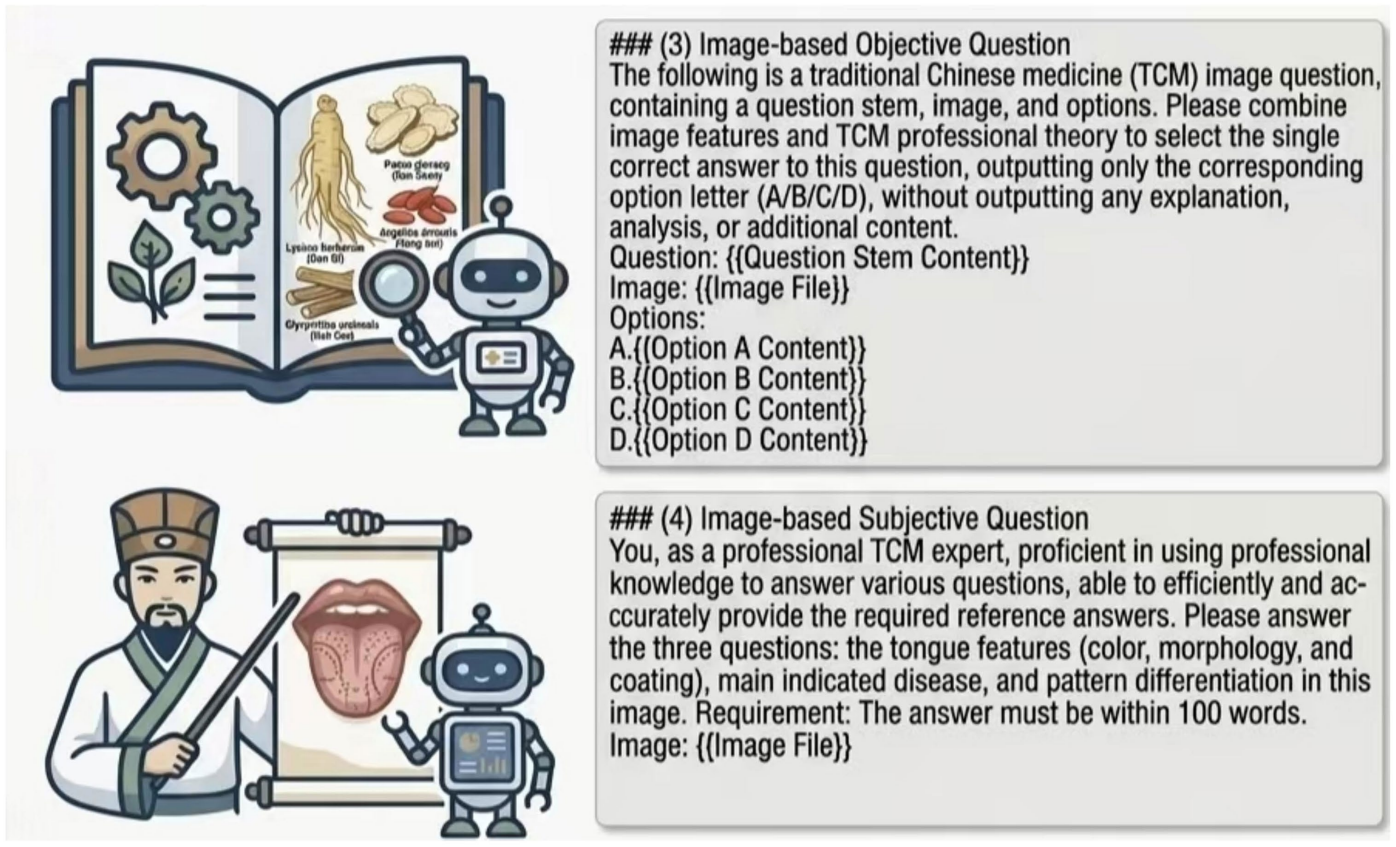
Design of image-based question prompts.

This study adopted an objective and neutral standardized prompt template, which was adapted to the specific requirements of textual and image-based question types, to avoid the confounding effect of prompts on model performance (11). The prompt for textual questions is shown in Figure 4, and the prompt design for image-based questions is presented in Figure 5.

#### 2.3.3 Quality control

1. *Repeated test validation*: For each question, independent repeated runs were performed 3 times for each model. The output consistent across at least 2 runs was adopted as the final valid result; if the output results were inconsistent across all 3 runs, the result was determined to be unstable.
2. Invalid output processing: The following scenarios were classified as invalid outputs and scored as incorrect: blank output, garbled code, completely irrelevant content, misinterpretation of core image features, output of only meaningless characters, and missing more than 2 of the 7 required dimensions in the response.
3. Model data inclusion criterion: If the proportion of invalid outputs of a model in a specific question type exceeded 5%, the evaluation data of the model in this module were excluded to ensure the validity of the overall evaluation dataset.

### 2.4 Evaluation criteria

#### 2.4.1 Objective questions evaluation Indicator

For textual objective questions and image-based objective questions, answer accuracy served as the evaluation metric (Equation 1). The degree of match between each model’s output and the official answer served as the evaluation criterion. Each correct answer received a point, while incorrect answers, missed answers, and answers with expression errors were all judged as invalid responses. We statistically analyzed the answer accuracy of each model across different knowledge modules, question difficulty levels, and cognitive hierarchies, to conduct a horizontal comparison of the overall performance of mainstream LLMs in the dimensions of TCM objective knowledge memorization, discrimination, and image recognition.


$$\text{Accuracy}=\frac{N_{\text{correct}}}{N_{\text{total}}}\times 100\%$$ (1)

#### 2.4.2 Subjective questions evaluation Indicator

1. *Automatic quantitative indicators*: Including BLEU-4 (Bilingual Evaluation Understudy-4) and ROUGE-L (Recall-Oriented Understudy for Gisting Evaluation-Longest Common Subsequence). BLEU-4 is based on N-gram co-occurrence and measures the precision of professional terminology matching between model outputs and reference answers. ROUGE-L uses the longest common subsequence to measure recall and precision, assessing the semantic coherence and completeness of model-generated answers relative to reference standards.
2. *Expert manual scoring*: Two TCM experts with at least a master’s degree and over 5 years of clinical and teaching experience independently scored the responses using a double-blind protocol. Model identifiers were masked to prevent experts’ subjective preferences from influencing the scores, and the Intraclass Correlation Coefficient (ICC) was used to verify inter-rater reliability (12).

For textual subjective questions, scoring was performed in accordance with the evaluation criteria of the National TCM Practitioner Qualification Examination (Practical Skills Test) across 7 dimensions: TCM disease diagnosis, TCM syndrome diagnosis, basis for disease and syndrome differentiation, differential diagnosis of similar syndromes, TCM therapeutic method, prescription name, and medicine composition, decoction and administration method. For image-based subjective questions, model outputs were scored on a 0–3 scale across three dimensions: completeness of tongue manifestation feature description, accuracy of primary disease judgment, and rationality of syndrome pattern differentiation.

#### 2.4.3 Error typology for qualitative analysis

To move beyond aggregate metrics and analyze model failures more systematically, we developed a four-category error typology informed by prior work on hallucination in natural language generation (10). E1 visual misidentification refers to incorrect recognition of morphological features in image-based tasks. E2 cross-modal association failure refers to correct or partially correct visual recognition but incorrect mapping to medicinal efficacy or clinical syndrome. E3 terminological-level error refers to fabrication of non-standard syndrome names or confusion between clinically similar but pathogenically distinct syndrome patterns, such as wind-heat versus wind-dryness invading the lung. E4 reasoning-level inconsistency refers to internal logical contradiction between syndrome differentiation, treatment principle, and herbal prescription within a single response. Two trained researchers independently coded a subset of incorrect responses using this typology, and disagreements were resolved through consensus; Cohen’s kappa was calculated to document inter-coder agreement.

### 2.5 Data analysis

The following statistical methods were used to analyze and compare model performance. All statistical tests were two-sided, with a significance level set at *α* = 0.05.

1. *Descriptive statistics*: The distribution characteristics of accuracy rates and manual scoring for each model were described using mean, standard deviation (SD), median, quartiles, and percentages. Data visualization was implemented using box plots, heatmaps, and bar charts.
2. *Overall comparison of multiple sets of dichotomous data*: Cochran’s *Q* test was used to compare overall correct response rates across models on objective questions and determine whether inter-model differences were statistically significant. If the overall test yielded *p* < 0.05, pairwise comparisons between groups were further performed using Bonferroni-adjusted paired McNemar’s tests.
3. *Nonparametric test for multiple related samples*: The Friedman rank-sum test was applied for the overall comparison of manual scoring on subjective questions across the 7 included models. If the overall test yielded *p* < 0.05, pairwise comparisons between groups were further conducted using the Wilcoxon signed-rank test, with the significance level adjusted by the Bonferroni method (13).
4. *Correlation analysis*: The Phi correlation coefficient was used to quantify the correlation of incorrect response distribution between different models.

## 3 Results

### 3.1 Overall performance of textual objective questions

#### 3.1.1 The overall accuracy rates of all models presented a distinct stepwise distribution

**Figure 6:**
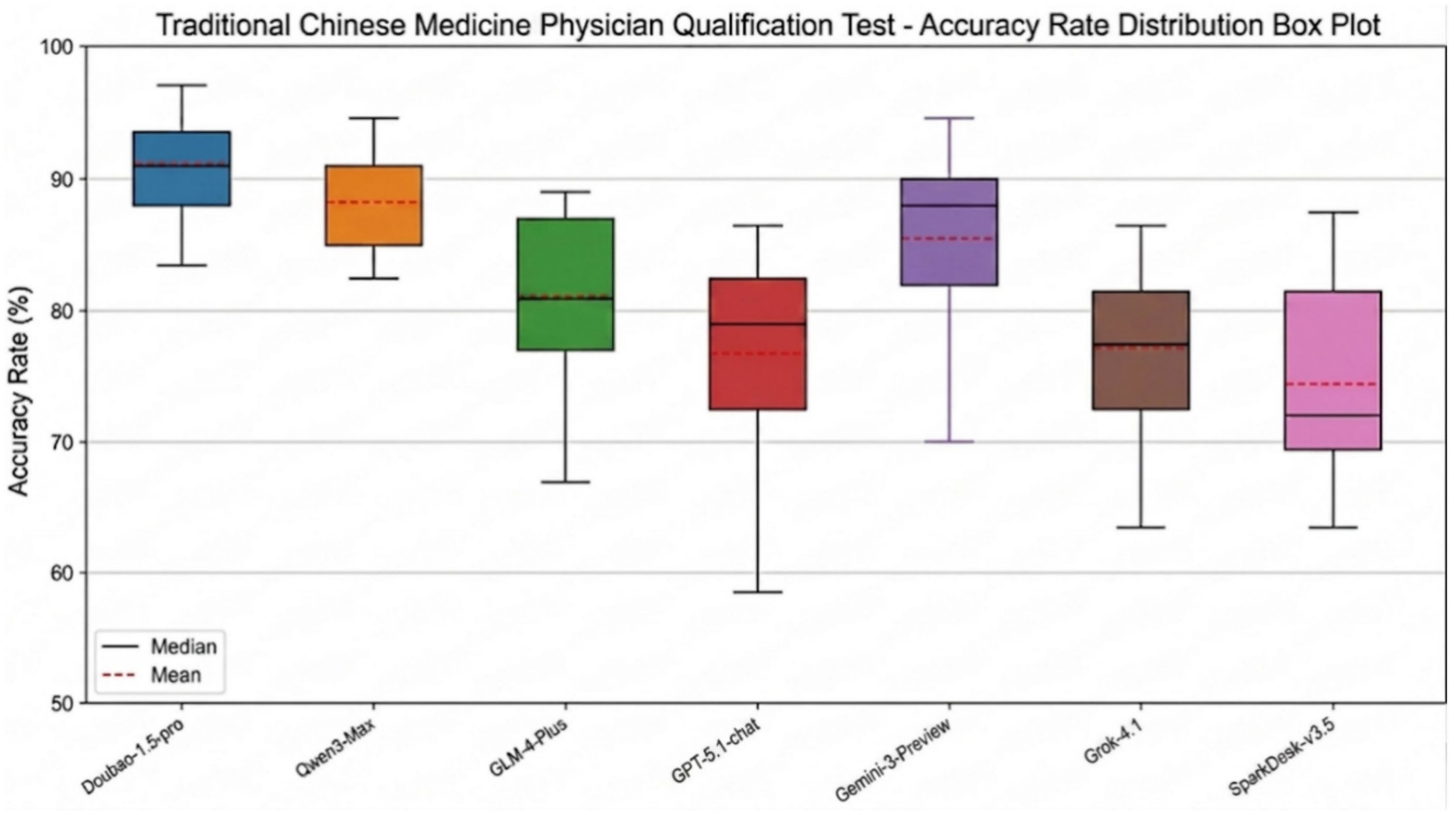
The overall accuracy distribution of the assessment model on objective questions in text.

Figure 6 presents the distribution of overall accuracy rates on textual objective questions across the 7 LLMs included in this evaluation. As shown in the figure, the overall accuracy rates of all models exhibit a distinct stepwise distribution.

Among them, the first-tier models achieved the best comprehensive performance, with all overall accuracy rates above 85%, namely Doubao-1.5-Pro (91.23%), Qwen3-Max (88.24%), and Gemini-3-Preview (85.42%). GLM-4-Plus had an accuracy rate of 81.02%, ranking at an intermediate level. In comparison, GPT-5.1-Chat, Grok-4.1, and SparkDesk-v3.5 exhibited relatively weaker response performance, with average accuracy rates of 76.66, 76.60, and 74.61%, respectively, indicating that their mastery of TCM objective knowledge still needs to be improved. Further Cochran’s *Q* statistical test was performed, and the results showed that there were statistically significant differences in the response accuracy rates on textual objective questions among the 7 LLMs (*p* < 0.001). Pairwise comparison results revealed that only the difference in accuracy rates between GPT-5.1-Chat and Grok-4.1 was not statistically significant, with all other pairwise comparisons reaching statistical significance.

An analysis of data dispersion and distribution, Doubao-1.5-Pro had a more concentrated score range, lower fluctuation amplitude, and the highest overall median value, presenting the optimal response stability and balance of knowledge mastery. In contrast, GPT-5.1-Chat, Grok-4.1, and SparkDesk-v3.5 had a wider accuracy range and higher degree of dispersion, indicating that these models had unbalanced knowledge mastery and relatively weak response stability.

#### 3.1.2 Models exhibited good mastery of core theoretical and clinical fundamental subjects, generally poor mastery of TCM classic knowledge

**Figure 7:**
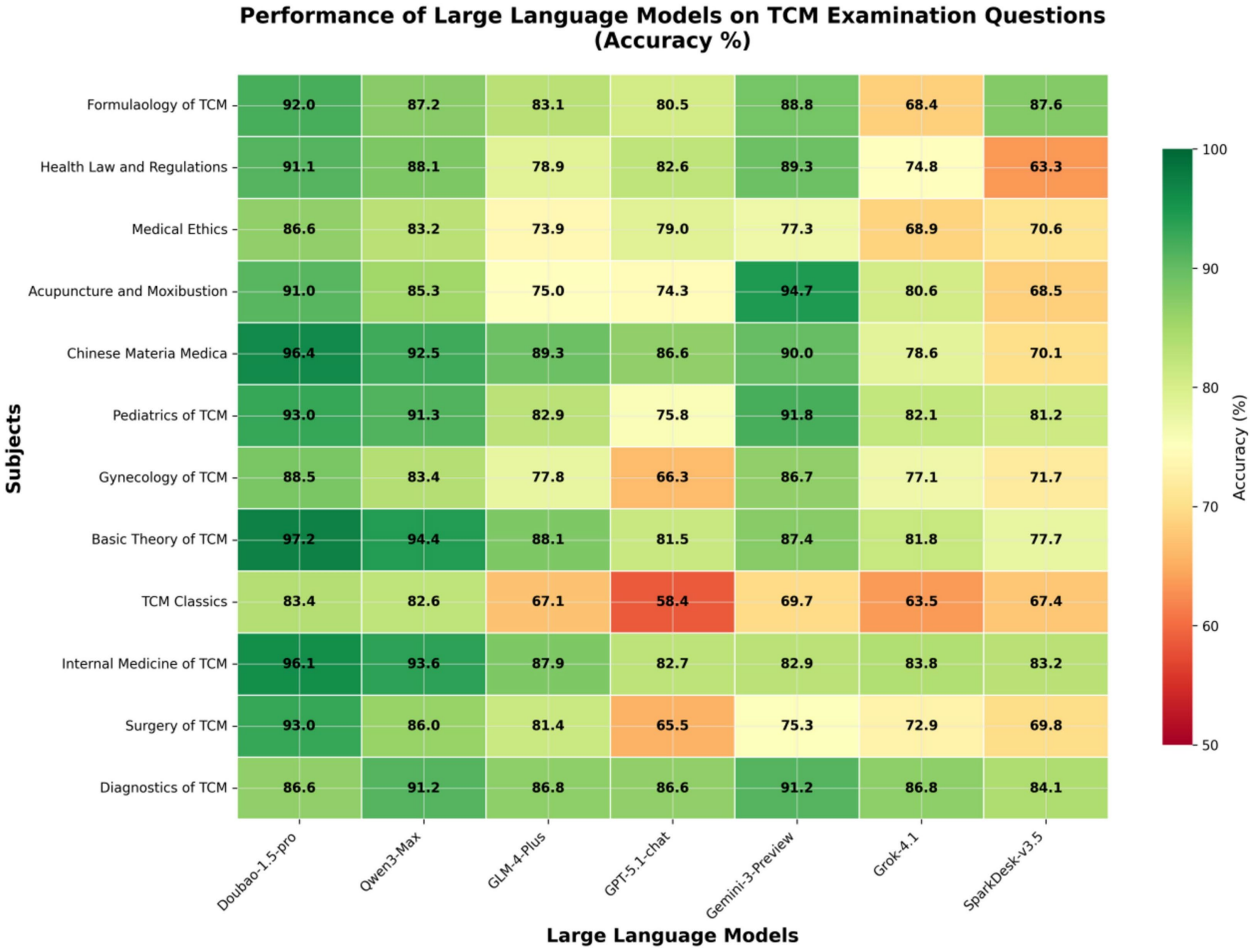
The accuracy rate distribution of the assessment model for different knowledge modules in objective questions of text-based tests.

Figure 7 presents the heatmap of accuracy rate distribution across the 12 subjects of the National TCM Practitioner Qualification Examination for each evaluated model. As shown in the figure, significant differences in performance were observed across different knowledge modules.

Among them, in core theoretical and clinical fundamental subjects represented by Basic Theories of TCM, TCM Internal Medicine, and TCM Diagnostics, the scores of all models were stably above 70%, with favorable overall performance. Taking Doubao-1.5-Pro with the best comprehensive performance as an example, its accuracy rate in the Basic Theories of TCM subject was as high as 97.15%, and the accuracy rates in Chinese Materia Medica and TCM Internal Medicine reached 96.36 and 96.06% respectively, demonstrating a solid foundation of TCM knowledge and strong memorization skills.

However, for subjects involving complex syndrome differentiation and specific diagnosis and treatment methods, such as Acupuncture and Moxibustion, TCM Surgery, and TCM Gynecology, significant differences were observed among models. For instance, the accuracy rates of GPT-5.1-Chat in TCM Gynecology and TCM Surgery were only 66.31 and 65.55%, which were significantly lower than its overall average accuracy level.

In addition, some models exhibited moderate performance in humanities, normative and professional literacy subjects represented by Health Regulations and Medical Ethics. For example, the accuracy rates of Grok-4.1 and SparkDesk-v3.5 in Medical Ethics were only 68.91 and 70.59%, significantly lower than those of other models. Such knowledge is time-sensitive and region-specific, with flexible formats, placing high demands on models’ contextual understanding and normative awareness.

In comparison, TCM classical knowledge proved to be a universal weakness across all evaluated models, with the overall accuracy rate significantly lower than that of other knowledge modules. Even for Doubao-1.5-Pro with the best comprehensive performance, its accuracy rate in the TCM Classics subject was only 83.43%, while the accuracy rate of GPT-5.1-Chat in this subject was 58.43%, which was the lowest value among all evaluation results across all subjects and models. TCM classics are written in classical Chinese, which presents a high comprehension threshold and considerable interpretive difficulty, reflecting that current mainstream LLMs still fall short in interpreting ancient TCM literature in depth. A representative example from our dataset vividly illustrates this challenge. The classical text describes when models were asked to identify the core pathogenic mechanism behind the synopsis of Shaoyin disease (少阴病) from the classic Shanghan Lun (Treatise on Cold Damage), the overall accuracy among the evaluated models plummeted to merely 14.3%. The classical text describes this condition as presenting with a ‘faint and fine pulse, with a constant desire to sleep’ (脉微细, 但欲寐也). Accurately answering this requires a deep structural understanding that these specific symptoms stem from ‘the deficiency of Heart and Kidney Yang, insufficiency of Yin and Blood, and the spirit losing nourishment’ (心肾阳虚, 阴血不足, 神失所养). While Doubao-1.5-Pro successfully deduced this connection, GPT-5.1-Chat incorrectly attributed the mechanism to ‘internal excess of Yin-cold with Yang failing to reach outward’ (心肾阳虚, 阴寒内盛, 阳不外达). It captured the Yang deficiency aspect but completely failed to comprehend the nuanced clinical relationship between the spirit’s lethargy (欲寐) and Yin-blood depletion. The widespread failure on such questions underscores that LLMs often rely on superficial keyword matching, struggling with the profound philosophical reasoning and systemic semantic integration required to truly decipher ancient medical texts.

#### 3.1.3 The accuracy rates of all models decreased significantly with increasing cognitive demand and question difficulty

**Figure 8:**
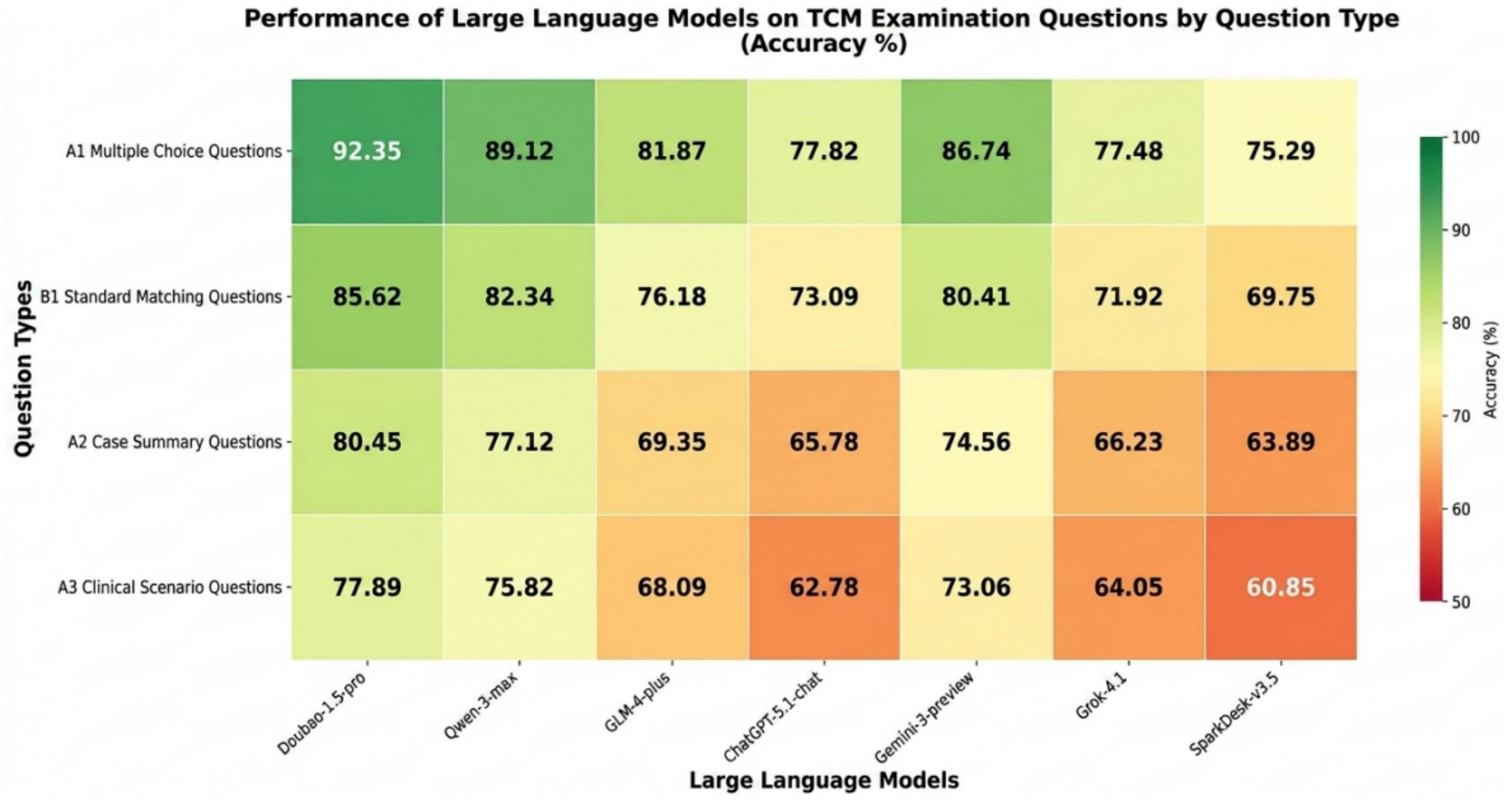
The accuracy rate distribution of the assessment model at different cognitive levels in objective questions of text-based tests.

Figure 8 presents the heatmap of accuracy rate distribution of each model across different cognitive hierarchies and question difficulty levels. As shown in the figure, the accuracy rates of all models decreased significantly as the assessment level of the questions shifted from lower-order memorization to higher-order clinical scenario application.

It should be noted that cognitive level and question difficulty are related but distinct constructs: cognitive level refers to the type of cognitive operation required (recall, application, analysis, synthesis), whereas question difficulty refers to the empirical proportion of correct responses. In our dataset, higher cognitive level questions tended to also exhibit higher difficulty, but the two are not interchangeable.

For lower-order cognition questions represented by Type A1 single-choice questions, all models achieved relatively high accuracy rates. Among them, Doubao-1.5-Pro ranked first with an accuracy rate of 92.35%, while Qwen3-Max and Gemini-3-Preview reached accuracy rates of 89.12 and 86.74%, respectively. Even SparkDesk-v3.5, which exhibited relatively weaker performance, maintained an accuracy rate of 75.29%, reflecting that mainstream LLMs have developed strong capabilities in basic TCM knowledge memorization.

For middle-order cognition questions represented by Type B1 standard matching questions, significant divergence was observed in the accuracy rates of the models. This type of question focuses on the discrimination and horizontal comparison of confusable knowledge points, requiring models to demonstrate knowledge association and discrimination skills. Among them, the three first-tier models still maintained a high accuracy rate of above 80%, while the accuracy rate of SparkDesk-v3.5 in the third tier dropped to 69.75%, with the performance gap between different tiers beginning to widen.

For higher-order cognition questions represented by Type A2 case summary questions and Type A3 clinical scenario questions, the accuracy rates of all models decreased significantly. Specifically, for Type A2 case questions, Doubao-1.5-Pro, which had the best comprehensive performance, only achieved an accuracy rate of 80.45%, a decrease of nearly 12 percentage points compared with Type A1 questions. The accuracy rates of models including GPT-5.1-Chat and SparkDesk-v3.5 dropped to 65.78 and 63.89%, respectively. For Type A3 clinical scenario questions that assess comprehensive clinical thinking, the accuracy rates of the models decreased further: Doubao-1.5-Pro achieved an accuracy rate of 77.89%, while the accuracy rates of GPT-5.1-Chat and SparkDesk-v3.5 were only 62.78 and 60.85%, respectively. This suggests that higher-order clinical reasoning and comprehensive analytical skills remain common weaknesses across current mainstream LLMs.

### 3.2 Overall performance of textual subjective questions

#### 3.2.1 Highly consistent tiered results between automatic and manual scoring

**Figure 9:**
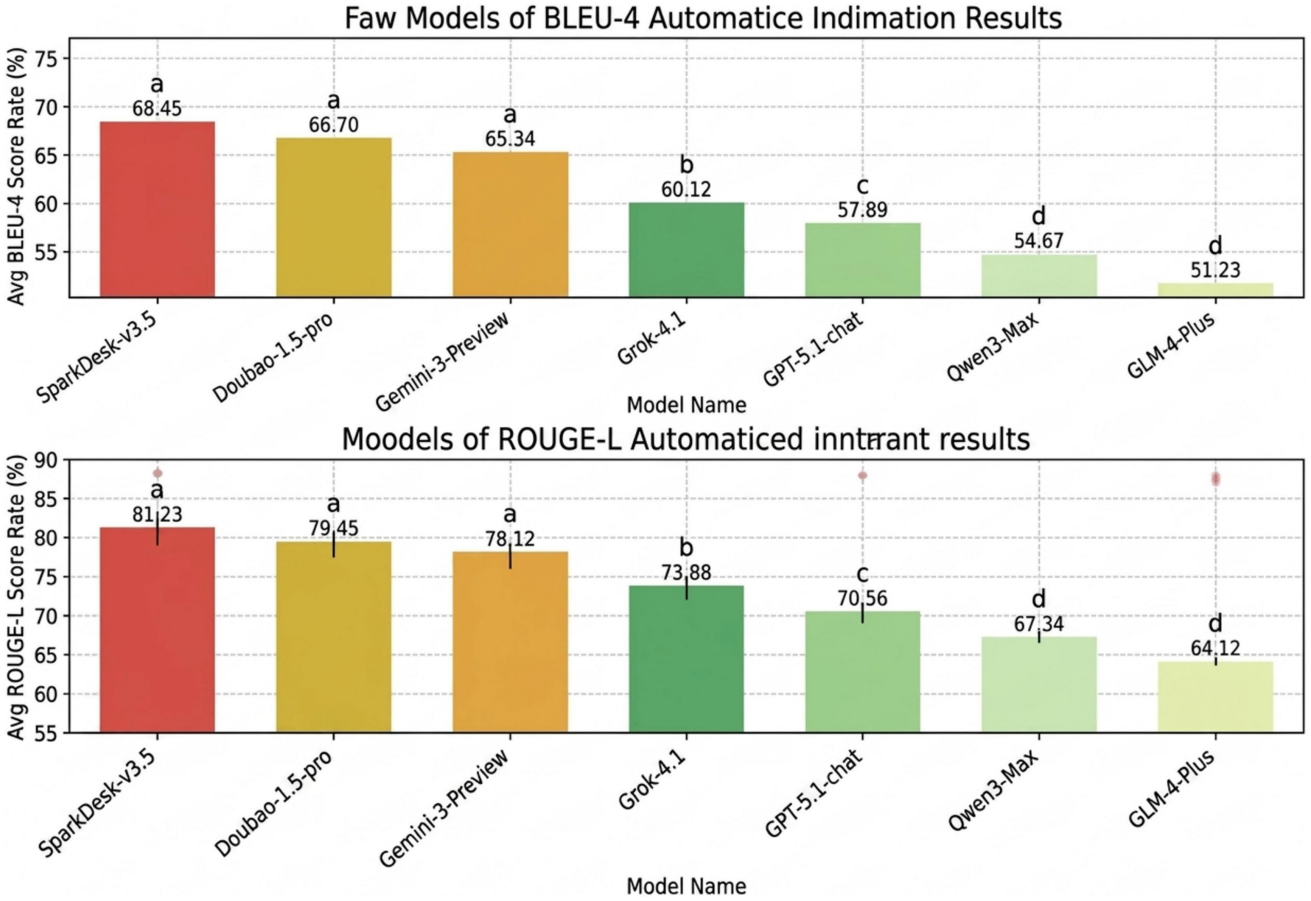
The automatic quantification results of the evaluation model in the subjective questions of the text.

BLEU-4 and ROUGE-L were used to automatically and quantitatively evaluate performance on textual subjective questions, and the results are illustrated in Figure 9. Obvious stepwise differences were observed across all models. The first-tier models included SparkDesk-v3.5, Doubao-1.5-Pro, and Gemini-3-Preview, all of which achieved a BLEU-4 score above 65% and a ROUGE-L score exceeding 75%.

In addition, the overall ROUGE-L scores of the models were approximately 13 percentage points higher than their BLEU-4 scores. This finding indicated that the models performed better in long-sequence coherence and overall expression fluency than in precise phrase-level matching. Such discrepancy is highly consistent with the characteristics of TCM medical case answers, which are usually lengthy and contain multi-layered logical reasoning for syndrome differentiation.

**Figure 10:**
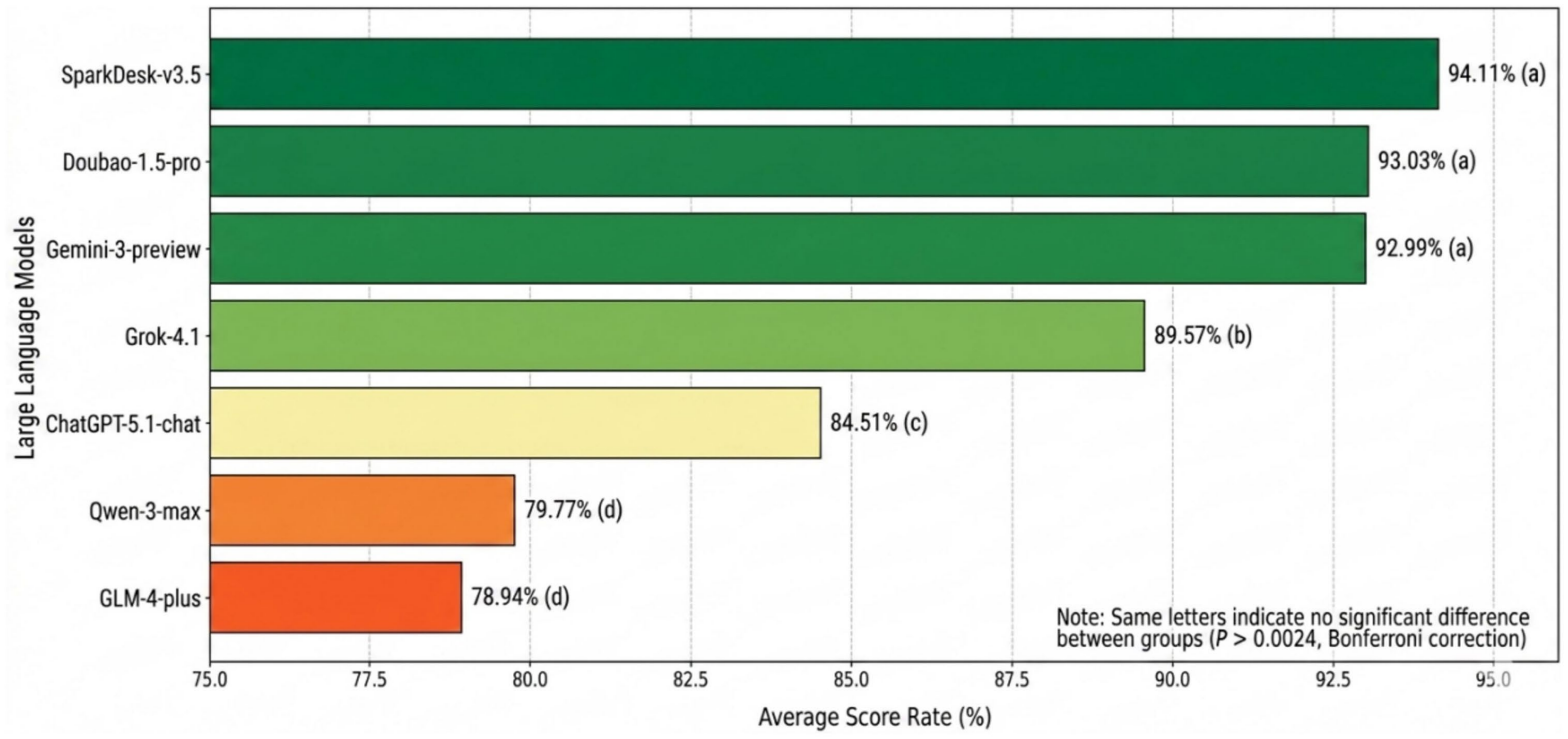
The expert manual scoring results of the evaluation model in the subjective text questions.

Further expert manual scoring was performed on the 7 included LLMs. Inter-rater reliability was verified using the Intraclass Correlation Coefficient (ICC). The result yielded an ICC of 0.86(95%CI:0.82–0.90), indicating good to excellent inter-rater reliability. Based on this consistent dataset, the average scoring rates are shown in Figure 10. The Friedman rank-sum test was applied for the overall comparison of the scoring results, which revealed statistically significant differences in the TCM syndrome differentiation and treatment capability among the models ( $X_{r}^{2}$ = 690, *df* = 6, *p* < 0.001). Further pairwise comparisons were conducted using the Bonferroni-adjusted Wilcoxon signed-rank test, and the results demonstrated that the outcomes of automatic quantitative indicators and expert manual scoring presented a highly consistent tiered structure.

#### 3.2.2 Performance advantage differences revealed by subjective vs. objective question results

Compared with the previously presented results of textual objective questions, the overall tiered structure of model performance on textual subjective questions changed considerably, indicating distinct competitive advantages across different LLMs.

The first tier consisted of SparkDesk-v3.5 (94.11%), Doubao-1.5-Pro (93.03%), and Gemini-3-Preview (92.99%), with all three models achieving an average scoring rate above 90%. No statistically significant differences were detected in pairwise comparisons within this group, which was therefore marked as tier “a.” Grok-4.1 belonged to the second tier, with a scoring rate of 89.57%. This model showed significant statistical differences when compared with all other models and was independently labeled as tier “b.” The third tier included GPT-5.1-Chat (84.51%), Qwen3-Max (79.77%), and GLM-4-Plus (78.94%), all with average scoring rates lower than 85%. Within this tier, GPT-5.1-Chat differed significantly from the other two models and was assigned to tier “c,” whereas Qwen3-Max and GLM-4-Plus exhibited no significant inter-group difference and shared the identical marker “d.”

Notably, several models exhibited pronounced discrepancies in performance between the two question types. SparkDesk-v3.5 achieved the highest scoring rate in the evaluation of textual subjective questions but yielded the lowest accuracy of only 74.61% for textual objective questions. By contrast, Qwen3-Max and GLM-4-Plus, which ranked high in objective question performance, fell into the lowest tier in subjective question assessment, with statistically significant gaps compared to the first-tier models.

The two question types differ substantially in assessment focus. Objective questions emphasize the memorization and discrimination of discrete knowledge points, whereas subjective questions place greater emphasis on the integrity of TCM syndrome differentiation logic, standardized expression of professional terminology, and comprehensive reasoning capacity. Consequently, the knowledge advantages demonstrated by certain models on objective tests did not translate into superior performance on subjective questions. The inherent strengths of individual models in text organization and logical expression were more fully reflected in subjective answer generation, ultimately producing results that diverged from the objective question rankings.

Representative qualitative analysis further revealed that several model failures were not random errors but reflected recurring patterns captured by the E1-E4 typology. Case 1 represents a terminological-level error (E3). In the fourth question of the TCM Practitioner Skill Examination, the patient’s presentation—dry cough, dry throat, scant and sticky sputum difficult to expectorate, with no fever—indicates wind-dryness invading the lung syndrome. Its core pathogenesis is dryness, manifested as dry cough, dry throat, and scant or sticky sputum. However, both GLM-4-Plus and GPT-5.1-Chat incorrectly diagnosed the case as wind-heat invading the lung syndrome, whose core pathogenesis is heat and is usually associated with yellow sputum, sore throat, and fever. This case exemplifies how models may confuse clinically similar but pathogenically distinct syndrome patterns.

Case 2 represents a reasoning-level inconsistency (E4). In a case of liver qi stagnation transforming into fire, one model correctly identified the syndrome as liver fire invading the lung but subsequently prescribed a formula intended for liver yang ascending. This created an internal contradiction between the diagnostic conclusion and the treatment strategy. Such cases indicate that professional-sounding answers may still contain clinically meaningful reasoning gaps, which is particularly important when AI outputs are used for teaching syndrome differentiation.

#### 3.2.3 Performance advantage differences revealed by subjective vs. objective question results

Compared with the previously presented results of textual objective questions, the overall tiered structure of model performance on textual subjective questions changed considerably, indicating distinct competitive advantages across different LLMs.

The first tier consisted of SparkDesk-v3.5 (94.11%), Doubao-1.5-Pro (93.03%), and Gemini-3-Preview (92.99%), with all three models achieving an average scoring rate above 90%. No statistically significant differences were detected in pairwise comparisons within this group, which was therefore marked as tier “a.”

Grok-4.1 belonged to the second tier, with a scoring rate of 89.57%. This model showed significant statistical differences when compared with all other models and was independently labeled as tier “b.”

The third tier included GPT-5.1-Chat (84.51%), Qwen3-Max (79.77%), and GLM-4-Plus (78.94%), all with average scoring rates lower than 85%. Within this tier, GPT-5.1-Chat differed significantly from the other two models and was assigned to tier “c,” whereas Qwen3-Max and GLM-4-Plus exhibited no significant inter-group difference and shared the identical marker “d.”

Notably, several models exhibited pronounced discrepancies in performance between the two question types. SparkDesk-v3.5 achieved the highest scoring rate in the evaluation of textual subjective questions but yielded the lowest accuracy of only 74.61% for textual objective questions. By contrast, Qwen3-Max and GLM-4-Plus, which ranked high in objective question performance, fell into the lowest tier in subjective question assessment, with statistically significant gaps compared to the first-tier models.

The two question types differ substantially in assessment focus. Objective questions emphasize the memorization and discrimination of discrete knowledge points, whereas subjective questions place greater emphasis on the integrity of TCM syndrome differentiation logic, standardized expression of professional terminology, and comprehensive reasoning capacity. Consequently, the knowledge advantages demonstrated by certain models on objective tests did not translate into superior performance on subjective questions. The inherent strengths of individual models in text organization and logical expression were more fully reflected in subjective answer generation, ultimately producing results that diverged from the objective question rankings.

### 3.3 Overall performance of image-based objective questions

Because the image-based objective dataset contained only 55 herbal identification questions, the findings in this subsection should be interpreted as exploratory rather than conclusive. The limited sample size and uneven distribution of herb categories constrain statistical power and preclude definitive category-level generalization.

Owing to the unstable performance of SparkDesk-v3.5 in image-based questions, this model was excluded from subsequent analysis. Only three domestic large language models (Doubao-1.5-Pro, Qwen3-Max, and GLM-4-Plus) and three international large language models (GPT-5.1-Chat, Gemini-3-Preview, and Grok-4.1) were included for comparative analysis, aiming to evaluate the capabilities of different models in two visual recognition tasks: Chinese herbal medicine identification and TCM tongue diagnosis.

#### 3.3.1 Image-based objective accuracy was significantly lower than textual objective accuracy

**Figure 11:**
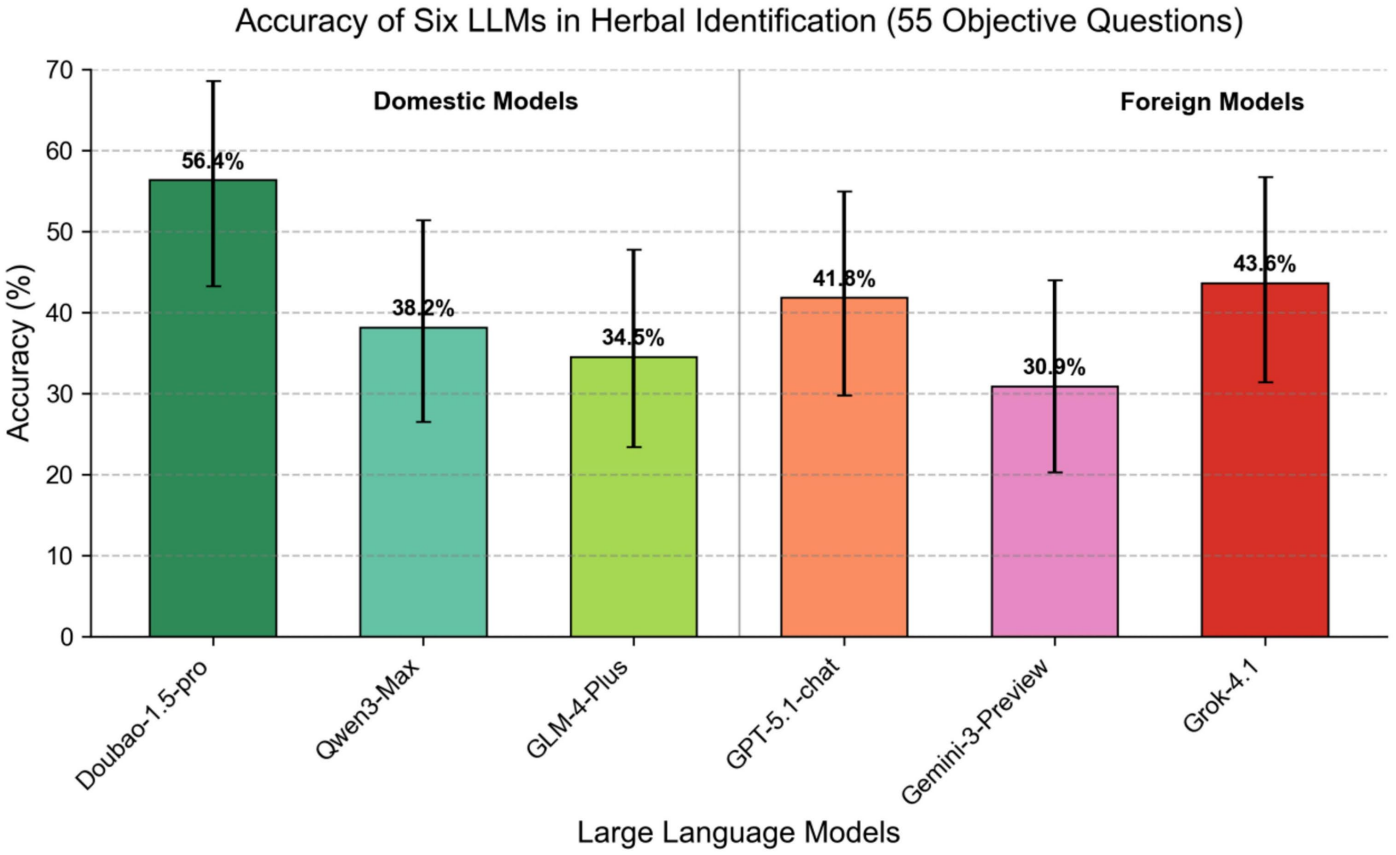
The overall accuracy rate of the assessment model in objective questions involving image recognition.

The evaluation results of image-based objective questions are presented in Figure 11. Compared with textual objective questions, all models exhibited an apparent decline in overall accuracy and generally weak performance in Chinese herbal medicine identification within this exploratory dataset. Among the included models, the domestic model Doubao-1.5-Pro achieved the highest accuracy at 56.4%. It was followed by the international models Grok-4.1 (43.6%) and GPT-5.1-Chat (41.8%). The accuracy rates of Qwen3-Max and GLM-4-Plus were 38.2 and 34.5%, respectively, while Gemini-3-Preview yielded the lowest accuracy of only 30.9%.

Overall, the best-performing domestic model appeared to maintain certain advantages in this image recognition task. Nevertheless, no model exceeded 60% accuracy, suggesting that current multimodal LLMs may still have substantial limitations in visual recognition of Chinese herbal medicines. Given the small sample size, this conclusion should be regarded as preliminary and requires validation in larger stratified datasets.

#### 3.3.2 Limitations in herbal morphological recognition and cross-modal knowledge association

**Figure 12:**
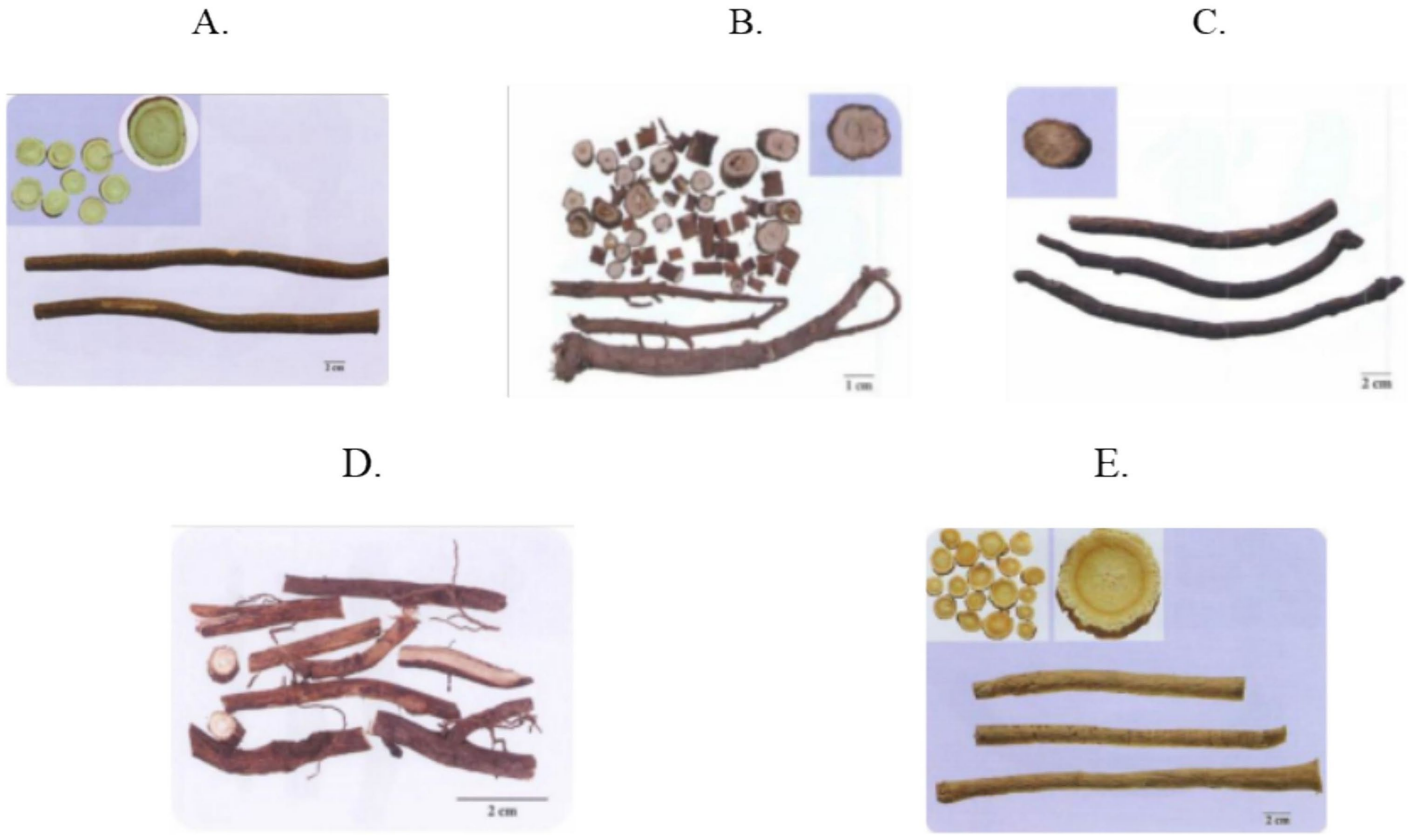
Example of objective questions based on image recognition 1. **(A)** Two long greenish-brown roots with circular cross-sections; **(B)** a thick brown root with multiple cut sections and an enlarged cross-section; **(C)** three dark slender roots with an inset cross-section; **(D)** reddish-brown root sections with circular cross-sections; and **(E)** pale yellow-brown roots with round slices and an enlarged cross-section.

**Figure 13:**
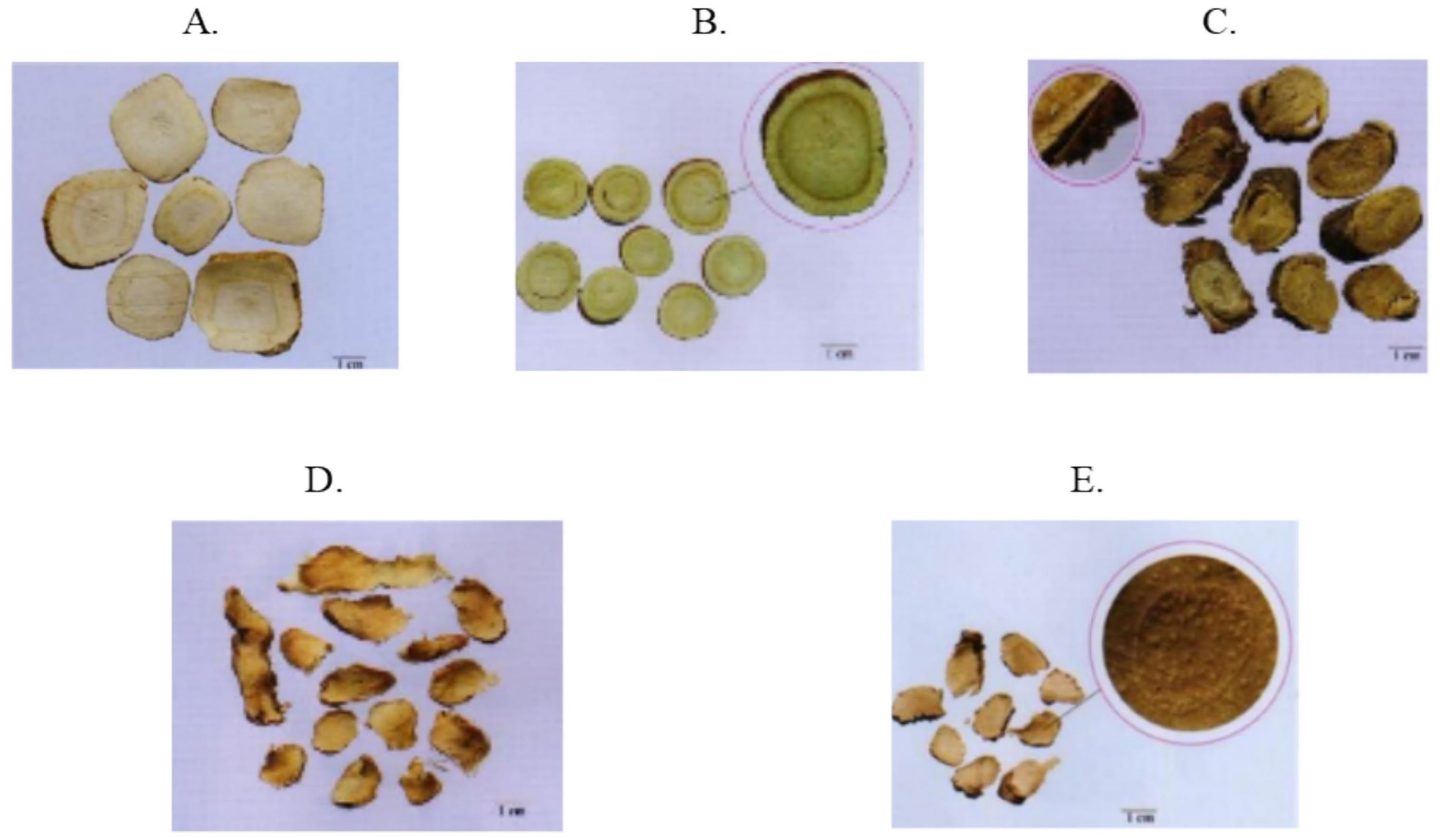
Example of objective questions based on image recognition 2. **(A)** Pale irregular root slices; **(B)** greenish-yellow circular slices with an enlarged cross-section; **(C)** dark-brown irregular pieces with an enlarged surface inset; **(D)** thin curled yellow-brown slices; and **(E)** small tan fragments with an enlarged view of brown powder.

Further exploratory analysis based on representative test questions (Figures 12, 13) revealed recurring errors in both morphological recognition of Chinese herbal decoction pieces and cross-modal knowledge association.

Consider Sample Question 1 from the image-based objective assessment. This item required the models to distinguish and identify root medicinal materials from multiple images of cylindrical herbal pieces with similar cross-sectional textures. Doubao-1.5-Pro, Qwen3-Max, and Grok-4.1 successfully identified the target herb and selected the correct Option C. In contrast, GLM-4-Plus and Gemini-3-Preview made incorrect judgments, choosing Option B and Option D, respectively. These findings suggest visual misidentification (E1): certain models failed to precisely capture subtle morphological characteristics of medicinal materials, such as radial cross-sectional textures, epidermis color differences, and unique wrinkled appearances. Deficient recognition of the specialized anatomical features of Chinese herbal medicines renders these models vulnerable to visual interference from morphologically similar specimens.

In Sample Question 2 of image-based objective tests, models were required to first identify herbal materials from images and then match them with their corresponding medicinal efficacies. None of the evaluated models selected the correct Option D, illustrating cross-modal association failure (E2) in the current dataset. When visual misidentification occurs at the recognition stage, subsequent reasoning about medicinal efficacy is compromised accordingly. These models struggled to complete the full workflow encompassing image recognition, knowledge retrieval, and logical deduction in complex clinical scenarios, falling short of the multimodal demands of professional TCM practice.

### 3.4 Overall performance of image-based subjective questions

The image-based subjective tongue diagnosis dataset contained 223 cases. Although this sample is larger than the herbal identification dataset, the findings should still be considered exploratory and should not be overgeneralized without further validation using larger and more diverse tongue-image datasets.

#### 3.4.1 Tongue diagnosis performance outperformed herbal medicine identification

**Figure 14:**
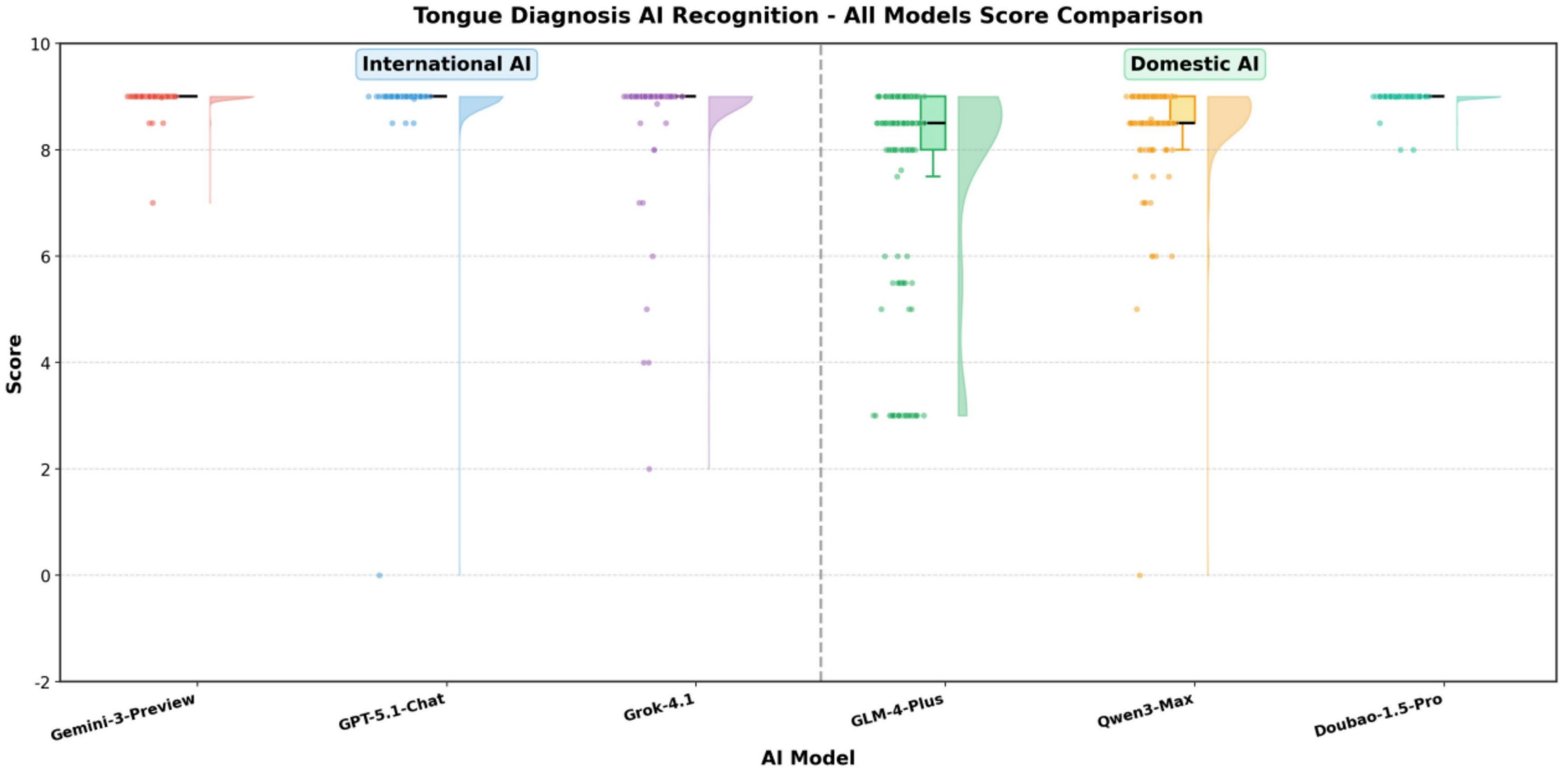
The distribution of expert scores in the assessment model for subjective image recognition questions.

In the tongue diagnosis task of image-based subjective questions, the models performed better than on the image-based herbal identification task described above within this exploratory dataset. As illustrated in the distribution of expert scores (Figure 14), Doubao-1.5-Pro (8.99 points), Gemini-3-Preview (8.98 points), and GPT-5.1-Chat exhibited the strongest performance, nearly reaching the full score of 9 points. Grok-4.1 (8.86 points) and Qwen3-Max (8.57 points) ranked next, while GLM-4-Plus obtained a relatively low score of 7.62 points.

#### 3.4.2 Models need improvement in capturing microscopic tongue features and holistic syndrome differentiation

Although the models achieved comparatively higher scores in subjective tongue diagnosis tasks than in Chinese herbal medicine identification, the evaluation results revealed important deficiencies in multimodal understanding. Firstly, the models lacked sufficient precision in capturing microscopic tongue manifestation features. Most models could accurately describe the general color of the tongue proper, as well as the thickness and distribution of tongue coating. However, they showed limited ability to identify subtle microscopic details, including dental indentations at the tongue margin, lingual fissures, petechiae and ecchymoses, and the moistness or dryness of tongue coating. Frequent omissions and misjudgments in description resulted in incomplete extraction of tongue manifestation information.

Secondly, there were notable differences among models in establishing logical correlations between tongue characteristics and TCM syndrome differentiation. For instance, in terms of the typical tongue manifestation of red tongue with scant coating, Doubao-1.5-Pro, Gemini-3-Preview, and Grok-4.1 accurately recognized its core nature of yin deficiency with internal heat and further diagnosed the corresponding syndrome as yin-deficiency common cold. In contrast, GLM-4-Plus presented biased interpretation of tongue color and coating properties, and incorrectly diagnosed this manifestation as wind-heat complicated syndrome, indicating cross-modal association failure (E2). In addition, several models misinterpreted professional terminology in complex scenarios involving combined tongue manifestations and multiple symptoms. As a typical manifestation of lung yin consumption syndrome, red tongue with scant coating accompanied by thready and rapid pulse was precisely linked to the essential pathogenesis of yin deficiency and fire hyperactivity by Doubao-1.5-Pro and GPT-5.1-Chat. Nevertheless, Qwen3-Max and GLM-4-Plus failed to identify the core pathogenesis of yin consumption induced by chronic diseases, thereby causing deviations in the interpretation of clinical diagnostic basis.

In summary, current multimodal LLMs have notable shortcomings in semantically aligning microscopic TCM tongue features (e.g., tongue color, coating property, and tongue morphology), professional terminologies, and clinical pathogenesis. Diagnostic biases may be particularly likely when comprehensive reasoning requires the combination of tongue manifestations and multi-source information such as clinical symptoms and physical constitution.

## 4 Discussion

This descriptive analysis demonstrates that while LLMs can generate structurally coherent text for TCM education, their performance exhibits significant heterogeneity across cognitive levels and modalities. Consistent with earlier studies highlighting the capacity of AI to process foundational medical knowledge (1, 2), our findings underscore the potential of top-tier LLMs in lower-order knowledge retrieval. However, our data also reveal a critical limitation: model factual accuracy, syndrome reasoning, and multimodal interpretation weaken considerably in complex, higher-order clinical scenarios.

### 4.1 Subjective-objective inversion in model performance

A notable finding of this study is the disconnection between how confident a model sounds and how accurate it actually is. For instance, SparkDesk-v3.5 ranked lowest in textual objective accuracy yet achieved the highest expert scoring and ROUGE-L metrics in subjective syndrome differentiation tasks. This discrepancy likely stems from the fundamental architecture of LLMs as probabilistic text generators (14, 15). They are excellent at mimicking the standard logic of TCM clinical records—moving smoothly from theory to treatment strategy and finally to the herbal formula. However, this professional-sounding exterior may conceal a weak factual foundation. The quality-readability-actionability framework for evaluating AI-generated health content helps explain this inversion: automated NLP metrics such as BLEU and ROUGE mainly capture readability and surface-level textual overlap, whereas expert scoring more directly reflects quality, clinical correctness, and educational actionability (16, 17). In medical education, students may therefore trust AI outputs because they appear structured and professional (18), potentially overlooking terminological-level errors (E3) or reasoning-level inconsistencies (E4), such as the wind-dryness versus wind-heat misdiagnosis case described above. Therefore, NLP metrics alone are insufficient for medical AI evaluation; human-in-the-loop clinical adjudication remains irreplaceable (19).

### 4.2 Multimodal failure and epistemological misalignment

Furthermore, our exploratory multimodal evaluations highlight a systemic bottleneck in current AI applications for TCM. No evaluated model exceeded a 60% accuracy threshold in Chinese herbal medicine identification in the limited image-based objective dataset (*N* = 55), and all exhibited difficulties in capturing microscopic tongue features in the tongue diagnosis dataset (*N* = 223). Rather than a simple issue of image resolution, this limitation may be attributed to a fundamental epistemological misalignment. Current multimodal foundation models are primarily pre-trained on Western-centric, standardized datasets characterized by distinct anatomical boundaries (20, 21). Conversely, TCM diagnostics rely heavily on holistic tacit knowledge, such as the dynamic assessment of a patient’s Shen (spirit) or subtle color gradations (22). In line with Kanjee et al. (23), vision models often fail when they lack specific clinical context. Our findings suggest that if students use these flawed AI tools without practicing with real specimens, they risk developing the wrong clinical instincts (24), which could interfere with their ability to learn traditional diagnostic skills.

### 4.3 Framework for TCM education

To safely integrate LLMs into TCM curricula, we propose a tiered, stepwise approach to AI integration. For foundational knowledge acquisition (A1/B1 levels), top-tier models (e.g., Doubao-1.5-Pro, Qwen3-Max, Gemini-3-Preview) can serve as virtual teaching assistants in flipped classroom settings. Students may consult LLMs before class for concept explanation, self-assessment, and retrieval of related knowledge points, thereby reducing extrinsic cognitive load (25). In Objective Structured Clinical Examination (OSCE) preparation, LLMs can supplement—but not replace—standardized patient encounters by providing instant feedback on students’ syndrome differentiation reasoning. For complex syndrome differentiation (A2/A3 levels), educators should adopt a critique-based method (26), tasking students with identifying and correcting purposefully flawed AI-generated medical records, such as the wind-dryness versus wind-heat misdiagnosis case. This approach is particularly suitable for clinical rotation debriefing sessions, where teachers can guide students to compare AI reasoning with authentic patient presentations. For teaching evaluation, LLM-generated responses can serve as formative assessment materials in which students are asked to identify embedded E1-E4 errors. Independent AI use in multimodal diagnostic tasks should remain limited until TCM-specific multimodal alignment technologies mature and robust safeguards against automation bias are implemented (27, 28).

## 5 Limitations

This study has several limitations. First, there is a substantial imbalance in sample sizes across evaluation modalities. While the textual objective dataset was robust (*N* = 7,903), the image-based objective dataset for Chinese herbal medicine identification was relatively small (*N* = 55), and the image-based subjective tongue diagnosis dataset was also limited (*N* = 223). This imbalance reduces statistical power, increases uncertainty around effect estimates, limits herb-category stratified analysis, and restricts the generalizability of all image-based findings. Therefore, image-based results should be interpreted as exploratory and preliminary rather than conclusive. Second, even the larger textual standardized datasets cannot fully represent the dynamic, multi-turn nature of real-world TCM consultations (29). In addition, the rapid iteration of LLMs means our findings represent only a current snapshot. Future research should focus on multi-turn dialogue assessments and the impact of long-term AI-assisted learning on medical students’ actual clinical competency.

## Data Availability

The raw data supporting the conclusions of this article will be made available by the authors, without undue reservation.
